# Patient-reported outcome measurement: a bridge between health and social care?

**DOI:** 10.1177/01410768211014048

**Published:** 2021-06-01

**Authors:** Sarah Hughes, Olalekan Lee Aiyegbusi, Daniel Lasserson, Philip Collis, Jon Glasby, Melanie Calvert

**Affiliations:** 1 Centre for Patient-Reported Outcome Research (CPROR), Institute of Applied Health Research Birmingham, University of Birmingham, Birmingham B15 2TT, UK; 2 National Institute for Health Research (NIHR) Applied Research Collaboration (ARC) West Midlands, University of Birmingham, Birmingham, UK; 3 Birmingham Health Partners Centre for Regulatory Science and Innovation, Birmingham, UK; 4 National Institute of Health Research (NIHR) Birmingham Biomedical Research Centre, University of Birmingham, University of Birmingham, Birmingham, UK B15 2TT; 5 University Hospitals Birmingham NHS Foundation Trust, Birmingham, UK B15 2GW; 6 Warwick Medical School, University of Warwick, Coventry, UK CV4 7HL; 7 Department of Social Work and Social Care, University of Birmingham, Birmingham, UK B15 2TT; 8 National Institute for Health Research (NIHR) Surgical Reconstruction and Microbiology Research Centre (SRMRC), Birmingham, UK

Social care is under increasing pressure globally. For economically developed countries, the cost pressures on long-term care services are set to grow as governments struggle to provide high-quality social care to an ageing population and a growing number of working age adults with complex needs.^1^ In England in 2018/2019, 841,850 people received publicly funded long-term social care, primarily in care/nursing homes or in their own home with a government spend of £22 billion.^2^ However, austerity has meant social care funding in real terms has fallen. Funding per person was at a lower level in 2019 than in 2010/2011, with an estimated funding gap of £1.5 billion. This funding shortfall, projected to be at least £2.7 billion by 2023/2024, means unmet need (i.e. people going without care and support) is a significant concern.^3^ Issues around workforce sustainability and a fragmented, means-tested social care system further challenge the delivery of social care.^4^ Healthcare, too, is confronted with the challenge of delivering safe, effective, quality care in the face of constrained budgets and, with COVID-19, a rapidly changing healthcare delivery landscape. As such, stakeholders across health and social care must give consideration to initiatives that will drive improvement in outcomes and quality of care.

Patient-reported outcome measures are questionnaires that capture an individual’s views on their physical, mental and social functioning, disease symptoms or health-related quality of life.^5^ Established within healthcare for over a decade, these measures patient-reported outcome measures are central to the delivery of person-centred care. At an individual level, patient-reported outcome measures supplement clinical assessments, facilitate referrals to specialist services, enhance patient–clinician communication, support decision-making around treatment, and assist with symptom checking and monitoring of disease progression. At an organisational level, patient-reported outcome measures are used to monitor provider performance, inform policy and guide quality improvement.^6^

In social care, patient-reported outcomes could be used similarly to support the delivery of person-centred care. Patient-reported outcome measures could promote choice and autonomy, ensuring care is responsive to a person’s wishes for their health and wellbeing. At a service level, patient-reported outcome measures could help identify unmet need, facilitate integration of health and social care, and guarantee that measures of quality emphasise person-centred outcomes (Figure 1).^7^

**Figure 1.**
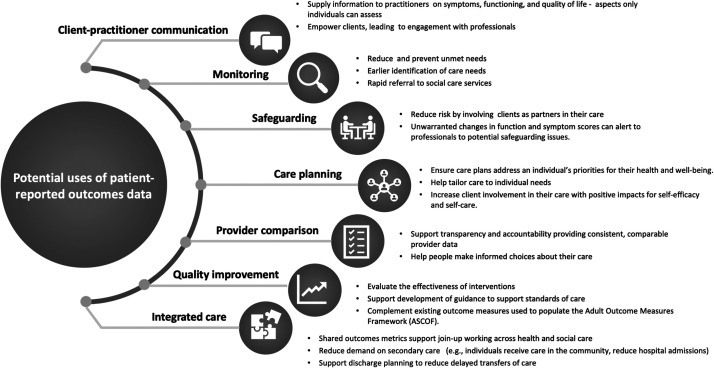
Potential uses of patient-reported outcomes data in integrated health and social care.

Consequently, patient-reported outcome measures may be viewed enablers of person-centred health and social care. However, their use in social care is not well documented.^8^ In this article, we explore the potential role of patient-reported outcome measures as a bridge between health and social care, clarifying current applications and future opportunities. We explore how cross-cutting patient-reported outcome measures could support better integration of health and social care services, better health outcomes, and enable increased independence and choice for people, as consumers of social care.

## Integrated care

‘Good health, healthcare and social care are mutually dependent and need to be approached together’.^9^ In England, fundamental differences in the delivery of health and social care contribute to disjointed working, resulting in delayed transfers from hospital, inappropriate placements, negative user experiences and poorer outcomes.^10^ Consequently, there have been calls for better integration, where healthcare and social care professionals work collaboratively to deliver coordinated care that emphasises prevention, supported self-care and provision of services closer to people’s homes.^11^ Patient-reported outcome measures are capable of supporting cross-service monitoring of functioning and disease symptoms. Standardising use could provide a common metric and a collective language of care that promotes inter-professional collaboration and effective coordination of services (Box 1).^5^

**Box 1.** Case study – David.

• David lives with long-term health conditions. He receives various payments that are intended to enable David to live independently and manage his own care and support. Through an agreed care plan, David receives ‘Direct Payments’ as part of a Personal Budget from his local authority and employs a Personal Assistant (PA) through a ‘brokerage service’ to support his personal care needs (e.g. activities of daily living, shopping). He also receives ‘Direct Payments’ through his Personal Health Budget from NHS continuing care funding via his local Clinical Commissioning Group. David uses this funding for training/therapies and equipment to meet his health and wellbeing needs as agreed through his healthcare support plan. He reports that ensuring his health and care needs are understood by his care teams and coordinating his support funds is challenging.
**Opinion**
• This case scenario highlights the current separation that exists between health and social care provision and the complexity of the funding system.^12^ David’s situation illustrates the need for social and healthcare provision to complement each other, as opposed to being treated as separate entities. Patient-reported outcome measures delivered by his social care team (e.g. social worker or PA) could help to identify changes in David’s health and wellbeing, triggering alerts to his healthcare team. Patient-reported outcome measures could also provide David with information that enables his views about his health to be conveyed clearly to his care teams. David could use this information to inform his decisions about his personal care, personal support and healthcare needs, helping him to maintain his independence and quality of life.

## Reducing and preventing unmet need in older, community-dwelling adults

Age UK estimated approximately 1.4 million older adults (65+ years) had unmet care needs in 2018.^13^ The King’s Fund suggest improved access to social care could be achieved if focus is shifted towards prevention by slowing the development of care and support needs.^4^ Predictors of unmet need (e.g. being an adult aged 50+ years, not having a long-standing illness, living alone) suggest many older adults with unmet needs may ‘fall under the radar’ of health and social care services.^11,14^ Generic patient-reported outcome measures or specific ‘needs-focussed’ measures (e.g. falls measures) could be used to identify unmet care needs. If deployed in partnership with GPs and primary care, electronic patient-reported outcome measures, embedded in digital health and care records, could be used for remote monitoring, alerting health professionals to potential problems and prompting timely referrals to health or social care services (Box 2).^15^

**Box 2.** Examples of patient-reported outcome measures in integrated health and social care.

**Use of patient-reported outcomes data in primary care to address unmet needs, support integrated care and support care planning/self-management**
• A UK mixed-methods qualitative study, utilising surveys (n = 100) and semi-structured interviews (n = 25) with GPs, found that 77% of GPs routinely used at least one patient-reported outcome measure to support screening/diagnosis or as an aid to clinical management. Results of the survey showed 38% of the sample used patient-reported outcome measures for chronic disease monitoring, 31% for care planning/self-management and 28% to support communication across different healthcare sector(s).^16^
**Using patient-reported outcomes data for symptom monitoring and reduction in need for additional care**
• A US randomised control trial compared the use of remote monitoring using electronic patient-reported outcome measures (i.e. reporting of 12 common symptoms and quality of life) via tablet computer to usual standard of care in cancer patients (N = 766) receiving routine outpatient chemotherapy. Compared with controls, researchers found the treatment group (i.e. patients who used the electronic reporting system) had fewer emergency admissions and fewer hospital visits, and stayed on chemotherapy for longer.^17^
**Symptom monitoring and disease tracking during COVID-19**
• Social care populations are particularly vulnerable to COVID-19.^18^ COVID-19 monitoring apps developed specifically for people living with frailty and their carers could prompt earlier identification (e.g. presentations with acute frailty syndromes as well as respiratory symptoms and fever), track disease progression and support recovery. Apps will need to be accessible and flexible to enable reporting by the 70% of care home residents with cognitive impairment.^19^ Patient-reported outcomes data can help understand how COVID-19 and its sequelae (i.e., Long COVID) affects this vulnerable group, where disease presentation is not yet well understood and decline in functioning may be an early indicator of disease. These data could guide treatment, for example, alerting care staff of the need for enhanced monitoring and earlier implementation of social distancing measures.

## Unmet need in residential settings

Measurement of patient-reported outcomes could also help address unmet need in care home settings where people are likely to be frail, older and have multiple long-term conditions.^19^ In planning care for residents, management decisions must be balanced between short-term interventions and longer-term disease control.^19^ Patient-reported outcome measures could support personalised care planning, ensuring decisions around care focus on health and wellbeing issues of greatest priority to the person. Proactive identification of unmet needs or early identification of deteriorating symptoms through the use of patient-reported outcome measures could ensure appropriate support, potentially arresting the need for additional care, including hospitalisation, at a later date (Box 2).

**Box 3.** Understanding the role of patient-reported outcomes measures in health and social care: directions for future inquiry.

• How are patient-reported outcomes measures used currently to support delivery of integrated health and social care?
• How are patient-reported outcome measures used currently within social care settings?
• What are the behavioural and attitudinal responses of stakeholders to the application of patient-reported outcome measures in social care settings?
• What are the barriers and facilitators to the implementation of patient-reported outcome measures in integrated health and social care?
• What patient-reported outcome measures are available currently to support the delivery of integrated health and social care and what is the quality of their measurement properties?
• How can practitioners utilise patient-reported outcomes measures to support the design and delivery of integrated health and social care interventions?

## Individualised care planning, safeguarding and palliative care

Patient-reported outcome measures facilitate prioritising of care aims that are congruent with a person’s wishes for their health and wellbeing.^20^ In this way, patient-reported outcome measures could promote individuals’ engagement in the care-planning process, and encourage independence and self-care (Box 2).^16^ Unwarranted changes in PROM scores could alert professionals to potential safeguarding concerns, triggering amendments to care plans to ensure the safety, effectiveness and efficacy of care.^8^ In palliative care settings, end-of-life measures could capture important information regarding autonomy, love, physical and emotional suffering, dignity, support and capability as part of preparations for supportive care at end of life and as a means of evaluating these interventions.^21^

## Patient-reported outcomes and pandemics

Patient-reported outcome measures, when used for tracking disease symptoms within the general population, provide vital data to understanding the epidemiology of new diseases such as COVID-19 and other threats to population health such as anti-microbial resistance.^22,23^ Monitoring could help clinicians and researchers understand disease presentation in social care populations where old age, frailty and co-morbidities are common and symptoms may be atypical (Box 2). Remote triage and diagnosis utilising patient-reported outcomes data could enable care to be managed safely in residential settings.

## Quality improvement and research

In England, the Adult Social Care Outcomes Framework focusses on four key outcomes social care (i.e. quality of life, safeguarding of vulnerable adults, reducing need for services and service satisfaction).^24^ It provides an established reporting structure into which patient-reported outcomes data could be incorporated. Such data could be used to support provider comparisons of social care at organisational, regional and national levels. Aggregate data could also facilitate service developments aimed at supporting integrated care. Augmenting client-reported social care outcome measures, patient-reported outcome measures could provide important health-related data to studies evaluating the effectiveness and efficacy of social care interventions, and the routine capture of patient-reported outcomes data in social care settings could inform research and quality improvement initiatives that cut across health and social care.^5^

## Challenges to the use of patient-reported outcome measures in integrated health and social care

Challenges will need to be addressed if patient-reported outcome measures are to be relevant to integrated care (Table 1). Health and social care organisations will need to consider how to integrate patient-reported outcome measures into existing workflows, consider system-wide and cross-system governance, and secure engagement from multiple stakeholders in both sectors. Careful consideration of candidate measures will be necessary to ensure instruments are relevant, reliable and valid. Next, we discuss some of these challenges and identify priority areas for future inquiry (Box 3).

**Table 1.** Challenges and potential solutions to use of patient-reported outcome measures across health and social care.

PROM selection and standardisation	• Given the large number of available measures, a comprehensive review of candidate patient-reported outcome measures and their psychometric properties is required to identify a pool of reliable and valid measures that are suitable for use.
	• Development of a Core Outcome Set of patient-reported outcomes, item bank (i.e. a database of validated questions) or the identification of a universal instrument to ensure a coordinated approach to data collection.
Accessibility	• Assess relevance and comprehensiveness of candidate measures for use with people who rely on social care services (i.e. content validity).
	• Evaluate candidate measures for ease of use (e.g. formatting, wording and readability, availability of multiple formats such as Braille and British Sign Language), establishing the reliability and validity of available formats.
Administration	• Flexible administration rules will be needed to accommodate individual needs without compromising validity (e.g. completion with support from a friend or family member or using alternative response formats).
	• Patient-reported outcome data may need to be supplemented with proxy reports. Patient-reported data should be given primacy over proxy measures.
Stakeholder engagement	• Agree a shared language around patient-reported outcomes. There are multiple terms for self-reported outcome measures used across health and social care (e.g. client-reported, patient-reported, participant-reported, person-centred coordinated care). These terms reflect the different models that inform the delivery of care, with potential to cause confusion, posing a barrier to effective communication between health and social care professionals.
	• Management teams should avoid mandating the use of patient-reported outcome measures without stakeholder engagement and support.
	• Supportive leadership must acknowledge practical challenges to implementation, provide appropriate resourcing and address attitudinal barriers sensitively.
	• Feedback to professionals should demonstrate how the collection of patient-reported outcomes have directly benefitted people with care and support needs and the delivery of their care.
	• Care team members should have access to relevant education and training on patient-reported outcome measures, and their applications.
System challenges	• The fragmented social care system and disjointed working between health and social case presents challenges to the use of patient-reported outcome data. Lessons learned from Adult Social Care Survey could help to promote implementation of patient-reported outcome measures in social care settings.
	• Information technology could support the use of patient-reported outcome measures in an integrated health and social care system.
	• Deployment of patient-reported outcome measures across health and social care brings additional costs to an already strained and underfunded system.

## Patient-reported outcomes, patient-reported outcome measures and models of care

In social care, the culture of care is informed by the social model of disability, where disability is considered to be caused by attitudinal, environmental and social factors within society that fail to take account of people with impairments and their associated needs. Healthcare, despite an increased emphasis on person-centred and value-based care, remains influenced by the medical model with its focus on impairment.^12^ Whereas terms such as ‘patient’ and ‘patient-reported’ are used commonly within healthcare, social care consumers rarely identify as ‘patients’ and, as such, terminology around patient-reported outcomes may be perceived as dissonant and incompatible by practitioners and people who use social care. For patient-reported outcome measures to be shared across health and social care, a shared, unambiguous, and culturally responsive language that facilitates communication across services will need to be adopted.

## Stakeholder engagement and support

Engagement by relevant stakeholders is vital if patient-reported outcome measures are to be deployed effectively across health and social care. In both sectors, it is important that service users, their loved ones and practitioners appreciate the benefits of the collection of patient-reported outcomes and are involved in decision-making around instrument selection and use.^25^ For buy-in from care staff, instrument selection must prioritise the person over service-level needs. Careful planning for implementation is essential, taking into consideration logistics, administrative burden, and evaluation and feedback processes. Leadership that endorses patient-reported outcome measures as part of an organisational culture of person-centredness and supports, in practical ways, the collection, analysis, and use of these data is fundamental.^26^ Leaders will need to acknowledge potential attitudinal barriers to patient-reported outcome measure uptake and ensure provision of education and training for professionals that promotes confidence and a greater understanding of patient-reported outcome measurement.^25^

## Patient-reported outcome measures Instrument selection and standardisation

Over the past three decades, hundreds, if not thousands, of patient-reported outcome measures have been developed. This surfeit of patient-reported outcome measures means it will be necessary to identify and select, in collaboration with stakeholders, valid and reliable instruments appropriate for use in the range of settings where integrated health and social care is provided. Social care is responsible for caring for people with a range of health conditions and multiple needs. Individuals may have low literacy, a learning disability, cognitive impairment and/or sensory disability that make it difficult for them to use patient-reported outcome measures.^27^ These factors make the selection of instruments challenging, both at the level of the individual and for services, where patient-reported outcome measures will need to be specific-enough to have good utility without being an administrative burden. Standardisation of measures will be key to encouraging use across settings and services.

## Accessibility to address health and care inequalities

Patient-reported outcome measures must be accessible if individuals are to accurately communicate information about their health. All too often, disabled people experience exclusion from the routine monitoring of their health and wellbeing afforded by patient-reported outcome measures.^27^ To ensure accessibility, questions must be relevant, question wording clear and formatting accessible. Innovative delivery solutions that exploit technological advances to increase inclusivity should be considered.^28^ The aims and benefits of completing a patient-reported outcome measure should be clear to respondents and administration flexible, so people get the help they need to engage with the reporting process.^27^ Practitioners must be sensitive to recognising when it may be appropriate (e.g. advancing cognitive decline) to supplement, not replace, patient-reported outcome measures with proxy-reported measures to ensure accurate representation of a person’s health and functioning.^29^

## Conclusions

Patient-reported outcome measures place the person at the centre of care. As measures of health, patient-reported outcome measures support people to live independent lives of their choosing. Importantly, patient-reported outcome measures provide a shared language of care, helping to bridge the gap between health and social care. The challenges of integrating patient-reported outcome measures across health and social care are significant; however, reporting frameworks and digital electronic patient-reported outcome platforms are already available and, following COVID-19, there is a growing need for safe and effective remote reporting of symptoms and interventions that encourage self-management. Efforts are now needed from stakeholders to grasp the opportunities patient-reported outcome measures offer to integrated care, so their benefits can be realised fully by the people who rely on these services.

## Supplemental Material

sj-pdf-1-jrs-10.1177_01410768211014048 - Supplemental material for Patient-reported outcome measurement: a bridge between health and social care? Click here for additional data file. Supplemental material, sj-pdf-1-jrs-10.1177_01410768211014048 for Patient-reported outcome measurement: a bridge between health and social care? by Sarah Hughes, Olalekan Lee Aiyegbusi, Daniel Lasserson, Philip Collis, Jon Glasby and Melanie Calvert in Journal of the Royal Society of Medicine

sj-pdf-2-jrs-10.1177_01410768211014048 - Supplemental material for Patient-reported outcome measurement: a bridge between health and social care? Click here for additional data file. Supplemental material, sj-pdf-2-jrs-10.1177_01410768211014048 for Patient-reported outcome measurement: a bridge between health and social care? by Sarah Hughes, Olalekan Lee Aiyegbusi, Daniel Lasserson, Philip Collis, Jon Glasby and Melanie Calvert in Journal of the Royal Society of Medicine

## Declarations

**Competing Interests:** SH is supported by the National Institute of Health Research (NIHR) Applied Research Centre (ARC), West Midlands. SH is company director, Narra Consulting Limited and undertakes consultancy work for Cochlear Ltd. MC is a National Institute for Health Research (NIHR) Senior Investigator and receives funding from the National Institute for Health Research (NIHR) Birmingham Biomedical Research Centre, the NIHR Surgical Reconstruction and Microbiology Research Centre and NIHR ARC West Midlands at the at the University of Birmingham and University Hospitals Birmingham NHS Foundation Trust, Health Data Research UK, Innovate UK (part of UK Research and Innovation), Macmillan Cancer Support, UCB Pharma. MC has received personal fees from Astellas, Takeda, Merck, Daiichi Sankyo, Glaukos, GSK and the Patient-Centered Outcomes Research Institute (PCORI) outside the submitted work. OLA is supported by the National Institute of Health Research (NIHR) Birmingham Biomedical Research Centre (BRC), West Midlands, Birmingham. OLA also receives funding from the Health Foundation and has received personal fees from Gilead Sciences. DL and PC have no conflicts of interest to declare. JG is a non-executive director of University Hospitals Birmingham, adjunct professor at Curtin University, member of the leadership team for an ESRC large grant on sustainable adult social care, and member of the expert advisory group for West Midlands ARC.

**Ethics approval:** Not applicable.

**Guarantor:** SEH

**Contributorship:** The idea for this article was conceptualised by MC, OLA, and DL. SEH prepared the initial draft of the manuscript. All authors provided critical feedback and approved the final version of the manuscript.
