# Supplementary figures and images for: Patient-reported outcome measurement: a bridge between health and social care?
Source: J R Soc Med. 2021 Jun 1;114(8):381–8. doi: 10.1177/01410768211014048 (PMC8358562; doi:10.1177/01410768211014048)

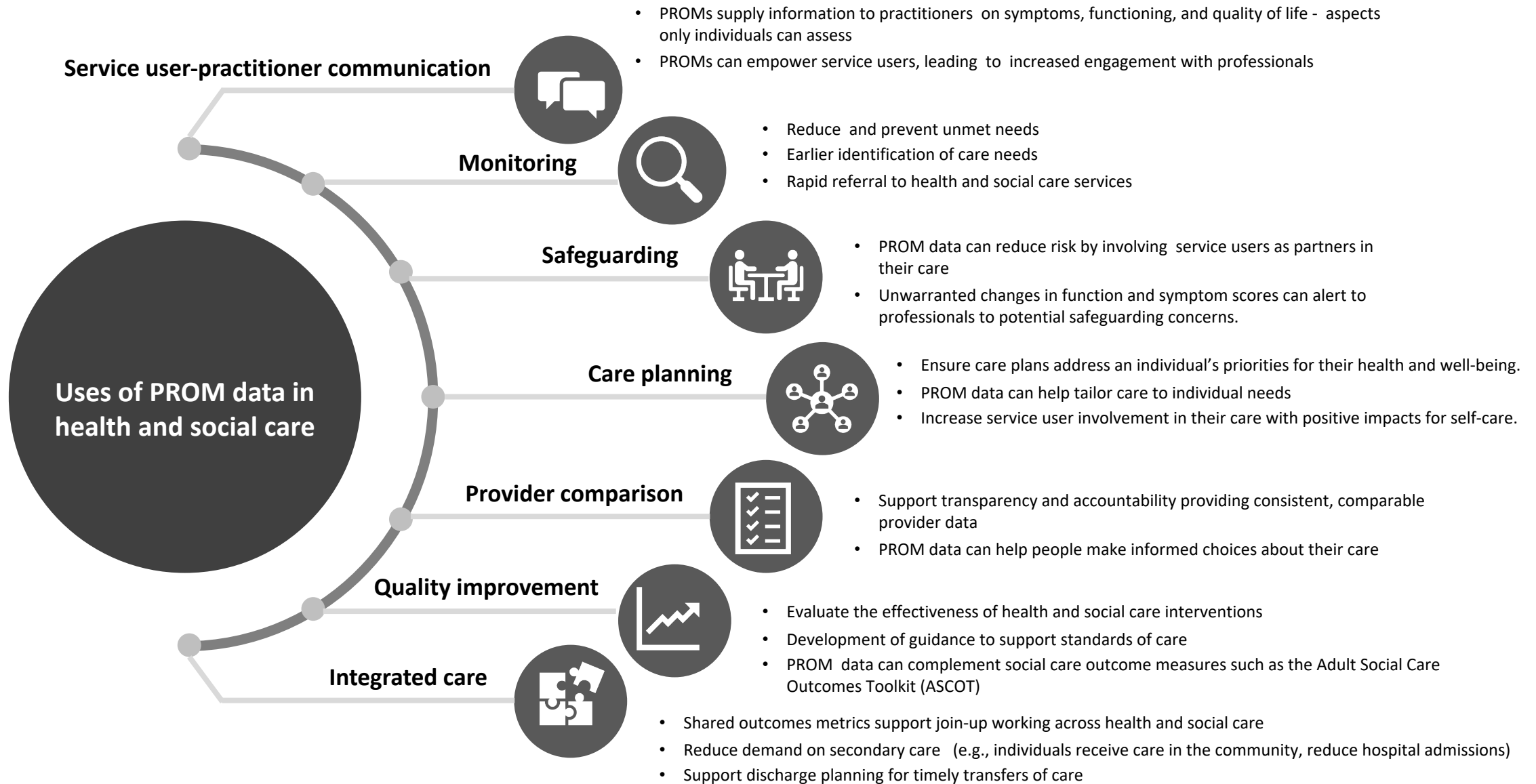

Supplement: sj-pdf-1-jrs-10.1177_01410768211014048 - Supplemental material for Patient-reported outcome measurement: a bridge between health and social care? [file sj-pdf-1-jrs-10.1177_01410768211014048.pdf]
