# Supplementary material for: Patient-reported outcome measurement: a bridge between health and social care?
Source: J R Soc Med. 2021 Jun 1;114(8):381–8. doi: 10.1177/01410768211014048 (PMC8358562; doi:10.1177/01410768211014048)
Supplement: sj-pdf-2-jrs-10.1177_01410768211014048 - Supplemental material for Patient-reported outcome measurement: a bridge between health and social care? [file sj-pdf-2-jrs-10.1177_01410768211014048.pdf]

From: ERourke@bmj.com

To: s.e.hughes@bham.ac.uk

CC: s.e.hughes@bham.ac.uk, O.L.Aiyegbusi@bham.ac.uk, D.S.Lasserson@bham.ac.uk, philip.collis@gmail.com, j.glasby@bham.ac.uk, m.calvert@bham.ac.uk

Subject: BMJ-2020-058999 Manuscript Decision Analysis

Body: --------------------

COVID-19: A message from BMJ: https://authors.bmj.com/policies/covid-19

18-Jul-2020,

Dear Mrs. Hughes,

BMJ-2020-058999 entitled "Patient reported outcomes measurement: A bridge between health and social care?"

Thank you for sending us this paper and giving us the chance to consider your work. We sent it out for external peer review and discussed it at the Analysis manuscript committee meeting (present: Sophie Cook, Peter Doshi, Emma Rourke).

After careful consideration we have decided not to publish your paper.

While we agreed that the topic was important, we did not feel that the article catered well for a general medical readership. The relevance to doctors caring for patients with social care needs didn't come across clearly enough. We also look for analysis articles to present a strong line of argument. We would have wanted authors to state their beliefs and explain how PROMs have the potential to support integration.

As you will appreciate we receive a large number of articles and often have to reject valuable and worthwhile work. When making an editorial decision we take the comments of the reviewers into account and also consider whether a piece will interest and inform our readers and whether it adds sufficiently to previous work. We have a large volume of analysis submissions competing for limited space at the moment and have had to make very difficult decisions about which papers to accept.

The reviewers' reports are available at the end of this letter.

Although The BMJ has an open peer review process, in which authors know who the peer reviewers were, we expect that you will keep the identity and comments of the peer reviewers for this paper confidential. You may, however, share the peer review comments in confidence (though not the names of the peer reviewers) with other journals to which you submit the paper. If you have any complaints about the peer review process or the conduct of the peer reviewers, please contact the editor who handled your paper. Please do not contact the peer reviewers directly.

You may want to consider sending your paper to another BMJ Journal, we have over 70 journals that cover a range of medical and clinical subject areas with the option to publish your paper open access; nearly half of our indexed journals rank within the top 10 of their category. A full list of the journals within the BMJ portfolio can be found here (https://journals.bmj.com/content/journals). If you would like any advice about where to send your paper, please contact BMJ's Transfer Editor (mrichards@bmj.com).

I'm very sorry for any disappointment caused and hope that the outcome of this submission does not deter you from future submissions to The BMJ.

Best wishes,

Emma Rourke
ERourke@bmj.com

Reviewer: 1

Recommendation:

Comments:
Thank you for the opportunity to review this paper. This article suggests that the use of patient-reports outcome measures at the interface of health and social care may support patient-centred care for improve care and patient outcomes.
As the BMJ is read by an international audience, I would suggest to define "social care" as this term may be specific to the UK healthcare system and to clarify differences between health and social care, and what is the intersection/interface between the two. It may also be worth noting what outcomes/outcome measures are being used (e.g. mortality, hospitalisation, cost?) to further justify the need for PROMs.
The definition and application of PROMs in healthcare settings are described clearly. Figure 1 provides a clear overview of examples of how PROMs may be implemented and used in health and social care.
With reference to the statement "standardised use of PROMs could provide a common metric and a collective language of care..." (Page 3); there are efforts to establish standardised outcomes (including PROMs) for research (COMET initiative), including for COVID-19 (as per one of the examples given). Would these be relevant in healthcare and social care settings, are there other efforts to standardised PROMs for health and social care? Consider also referencing COSMIN https://www.cosmin.nl/
The challenges and potential solutions described in Table 1 seem comprehensive.
Another point of discussion to consider is embedding PROMs in registries.
I think the number of PROMs is in the order of thousands not hundreds, based on the various systematic reviews of PROMs.
Box three provides a research agenda. Could PROMs also have a role in helping to identify and design social care interventions, for example to address important outcomes to the community/patient population?
Overall the article is well written and provides a good discussion about the benefits of implementing PROMs in health/social care, the challenges and potential solutions.

Additional Questions:
The BMJ uses compulsory open peer review. Your name and institution will be included with your comments when they are sent to the authors. If the manuscript is accepted, your review, name and institution will be published alongside the article.

If this manuscript is rejected from The BMJ, it may be transferred to another BMJ journal along with your reviewer comments. If the article is selected for publication in another BMJ journal, depending on the editorial policy of the journal your review may also be published. You will be contacted for your permission before this happens.

For more information, please see our peer review terms and conditions.

Please confirm that you understand and consent to the above terms and conditions.: I consent to the publication of this review

Reviewer: 2

Recommendation:

Comments:
Thank you for the opportunity to review this manuscript. This is an important topic and a timely paper. My perspective on this is as a facilitator of patient involvement in research priority setting, so I am a strong supporter of the notion of PROMs - and I was pleased to see a patient partner was involved in the production of this paper. Nevertheless, while I support projects in the health and social care sectors, I am not a practitioner delivering services, nor designing or commissioning them. I am therefore conscious that some

aspects of my review may not be relevant as I am probably not your intended audience. My comments/observations are as follows:

The authors present a compelling context for the use of PROMs across both health and social care, setting out their use, value and relevant a) for an ageing population and b) within the COVID-19 pandemic.

I wondered if it would be useful to include an practical, real-life example of what PROMs data looks like - eg a form in which the data is collected, or an example of how it would appear on a patient's record.

Case study - David: is there scope to say more about why this situation is challenging? There's a good description of why the use of PROMs data could make life easier for David, but it might be helpful to describe the negative consequences of the current challenges being left unaddressed, both for David and the people working with him.

From page 4 onwards I was wondering how the data would typically be collected, by whom, in what way. Maybe this isn't relevant to the intended audience, but would a care worker reading this understand the practicalities and how routine PROMs collection/use would fit into their job?

The section on end of life measures really brought it to life for me.

It was useful to see the challenges and solutions set out in table 1, and the directions for future enquiry in Box 3 were clear. I was struck by the potential barriers around language (page 10, line 50) and attitudes (page 11, line 13) and would be interested to know more about this. The final sentence of the conclusion is a call to action. However, I wasn't entirely sure who this is aimed at. Who is best placed to take this challenge on, or to coordinate or be part of a coordinated response? That part wasn't clear to me.

I hope these comments are helpful.
